# ﻿Systematic review of the firefly genus *Emeia* Fu, Ballantyne & Lambkin, 2012 (Coleoptera, Lampyridae) from China

**DOI:** 10.3897/zookeys.1113.79721

**Published:** 2022-07-18

**Authors:** Cheng-Qi Zhu, Xiao-Dong Xu, Ying Zhen

**Affiliations:** 1 College of Life Sciences, Zhejiang University, Hangzhou, Zhejiang, China Westlake University Hangzhou China; 2 Westlake Laboratory of Life Sciences and Biomedicine, Key Laboratory of Structural Biology of Zhejiang Province, School of Life Sciences, Westlake University, Hangzhou, Zhejiang, China Zhejiang University Hangzhou China; 3 Institute of Biology, Westlake Institute for Advanced Study, Hangzhou, Zhejiang Province, China Westlake Institute for Advanced Study Hangzhou China

**Keywords:** Cytochrome c oxidase subunit I, DNA barcoding, *Emeia*, firefly, Lampyridae

## Abstract

The Luciolinae genus *Emeia* Fu, Ballantyne & Lambkin, 2012 is reviewed. Phylogenetic relationships based on *cox1* DNA barcoding sequences from 42 fireflies and 2 outgroup species are reconstructed. The dataset included three main Lampyridae subfamilies: Luciolinae, Photurinae and Lampyrinae, and *Emeia* was recovered within Luciolinae. A new species, *Emeiapulchra* Zhu & Zhen **sp. nov.**, is described from the wetland of Lishui, Zhejiang, China. *Emeiapulchra* is sister species to *E.pseudosauteri* from Sichuan, which is supported by morphological characters and a phylogeny based on DNA barcoding sequences. The two species are separated geographically as shown on the distribution map. A key to species of *Emeia* using males is provided.

## ﻿Introduction

*Emeia* Fu, Ballantyne & Lambkin, 2012 (Luciolinae) was established as a monotypic genus (Fu et al. 2012) with *Emeiapseudosauteri* (Geisthardt 2004) as the type species. *Emeiapseudosauteri* was first described from Mount Emei, Sichuan, China by Michael Geisthardt in the genus *Curtos* Motschulsky, 1845 (Geisthardt 2004), and then transferred to *Emeia* based on morphological evidence (Fu et al. 2012). The genus *Emeia* Fu, Ballantyne & Lambkin had only one species (*E.pseudosauteri*) recorded in China before this study. The primary phenotypic feature of *Emeia* was the trilobite-like larva. The thoracic and abdominal terga of *Emeia* larvae are distinct. The lateral thoracic tergal margins are broad, similar to those of a trilobite “cephalon”, while the abdomen is narrow and curls ventrad in the posterior part. At present, definition of the genus *Emeia* is based on the morphology of *E.pseudosauteri*, which makes it insufficient in light of the discovery of a second species.

In this study, based on specimens collected from Lishui, Zhejiang, China, we describe adults of *Emeiapulchra* Zhu & Zhen sp. nov. based on morphological and molecular data. We compare it with the previously described *E.pseudosauteri*. We also provide new information on the adult male hind wing venation of the type species *E.pseudosauteri*. With our detailed examination of both species, we present a systematic review of the genus *Emeia* and a key to species.

## ﻿Materials and methods

### ﻿Abbreviations

**EL** elytral length;

**EW** elytral width;

**PL** pronotal length;

**BL** body length (the sum of PL, EL and the length of the exposed portions of the head from the pronotum);

**BW** body width (the greatest distance across the elytra, BW=2EW);

**T7, 8** abdominal tergite numbers;

**V6, 7** abdominal ventrite numbers.

Adult males of *Emeiapulchra* Zhu & Zhen sp. nov. were collected from Jiulong National Wetland Park, Lishui, Zhejiang Province in April, 2020. The holotype and paratypes of the new species are stored at School of Life Sciences, Westlake University, Hangzhou, Zhejiang. Samples of both male and female *Emeiapseudosauteri* were collected from Mt. Tian Tai, Sichuan Province in April, 2021.

**Figure 1.**
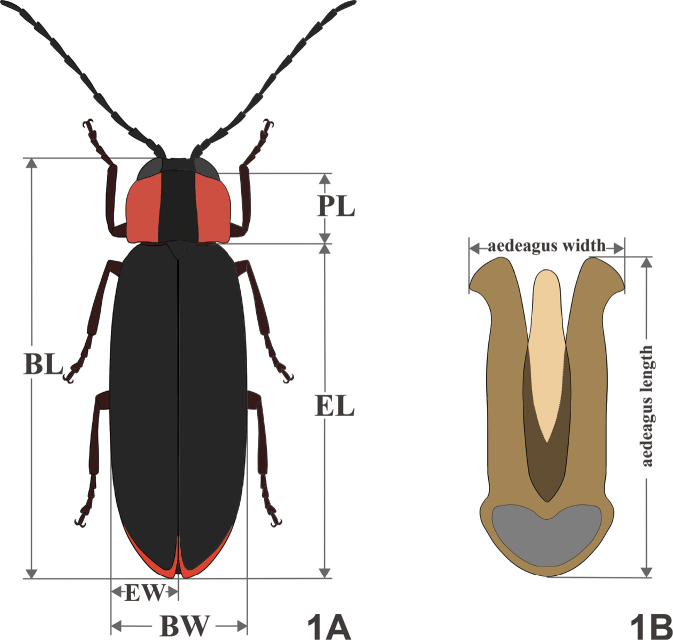
Measurement methods and terminology **A** male habitus, dorsal view **B** aedeagus, ventral view.

Habitus images were taken using a Nikon D7500 camera. Images of genitalia were taken using a Nikon D7500 camera mounted on an SZ650 microscope (Chongqing Optec Instrument Co., Ltd.) under reflection or transmission light. Images were edited using Adobe Photoshop CS6. Morphological terminology and measurements follow those decribed in Douglas (2017). The body length (**BL**) is the sum of the pronotal length (**PL**) and elytral length (**EL**) plus the length of the exposed portions of the head from the pronotum. The abbreviations **EW** and **BW** (**BW=2EW**) denote elytral width and body width, respectively (Fig. 1A). The length and width of the aedeagus and aedeagal sheath were measured under the microscope using the OLYMPUS cellSens Dimension software (v 3.1.1) (Fig. 1B). The dissected aedeagus and aedeagal sheath structures are preserved in pure glycerol in small vials with the corresponding specimens.

We sequenced the *cox1* gene barcode fragment from *Emeiapseudosauteri* and *E.pulchra*. Specifically, total DNA of the two *Emeia* species was isolated using the DNeasy Blood and Tissue Kit (Zhejiang Easy-Do Biotech CO., Ltd.), according to the manufacturer’s protocol. The primers LCO 1490 and HCO 2198 (Folmer et al. 1994) were used to amplify the barcode fragments of the mitochondrial gene cytochrome c oxidase subunit I (*cox1*). We performed the PCR reaction in a 25 μL reaction mix containing 1× PCR buffer, 1 μL of each primer in a final concentration of 1 μM, 1 μL of template, 0.2 mM of each dNTP and 0.5 units of Taq polymerase (Takara Biomedical Technology CO., Ltd). The PCR thermal regime consisted of an initial denaturation at 95 °C for 3 min; 30 cycles of 30 s at 94 °C, 30 s at 48 °C and 30 s at 72 °C, followed by a 5 min final extension at 72 °C. PCR products were checked by electrophoresis in 1% agarose gel at 170 V for 20 min, and visualized under a UV transilluminator with nucleic acid dye (Cofitt Life Science, Hong Kong). The PCR products were cleaned using Easy Gel Extraction & Clean-up kit (Zhejiang Easy-Do Biotech CO., Ltd.). The cleaned products were sequenced with an ABI 3730XL sequencer (Applied Biosystems, California, USA) by Zhejiang Sunya Biotechnology Co., Ltd.

**Figure 2.**
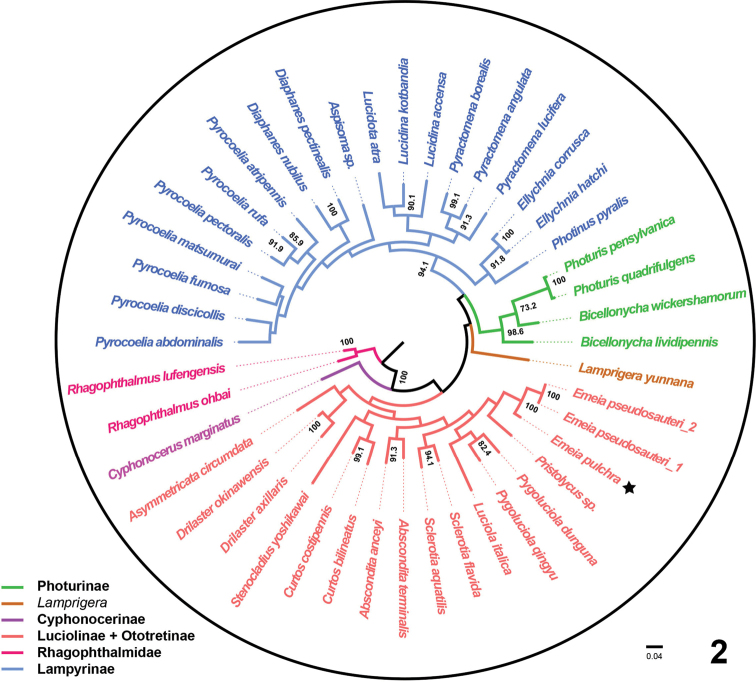
Maximum likelihood *cox1* gene tree of *Emeia* and related genera. The star highlights the new species, *E.pulchra* Zhu & Zhen sp. nov. *Emeiapseudosauteri*_1 was downloaded from GenBank (MN722654.1). *Emeiapseudosauteri*_2 was sequenced during this study. Bootstrap values greater than 0.7 from 1000 replicates are shown.

MEGA6 (Tamura et al. 2013) was used for phylogenetic reconstruction. *Cox1* barcode sequences from three main subfamilies, *i.e.*, Luciolinae, Photurinae and Lampyrinae, were included, and sequences from the family Rhagophthalmidae were used as an outgroup (Table 1). The maximum likelihood method was used with 1000 bootstrap replicates (Fig. 2). The phylogenetic relationships were displayed using iTOL (v6; https://itol.embl.de/). The new *cox1* sequences from *Emeia* have been deposited in GenBank (accession numbers OK144132 and OK103803).

**Table 1.** Genbank accession numbers for *cox1* sequences used for the phylogenetic analysis.

Species	Family	Sub-family	GenBank id
*Pyrocoeliapectoralis*	Lampyridae	Lampyrinae	KP763467.1
*Pyrocoeliarufa*	Lampyridae	Lampyrinae	AF452048.1
*Pyrocoeliaabdominalis*	Lampyridae	Lampyrinae	AB608766.1
*Pyrocoeliaatripennis*	Lampyridae	Lampyrinae	AB608767.1
*Pyrocoeliadiscicollis*	Lampyridae	Lampyrinae	AB608768.1
*Pyrocoeliafumosa*	Lampyridae	Lampyrinae	AB608769.1
*Pyrocoeliamatsumurai*	Lampyridae	Lampyrinae	AB608770.1
*Diaphanesnubilus*	Lampyridae	Lampyrinae	MG200080.1
*Diaphanespectinealis*	Lampyridae	Lampyrinae	NC_044793.1
*Photinuspyralis*	Lampyridae	Lampyrinae	KY778696.1
*Ellychniacorrusca*	Lampyridae	Lampyrinae	KR483038.1
*Ellychniahatchi*	Lampyridae	Lampyrinae	JF887410.1
*Pyractomenalucifera*	Lampyridae	Lampyrinae	MF640134.1
*Pyractomenaborealis*	Lampyridae	Lampyrinae	HQ928227.1
*Pyractomenaangulata*	Lampyridae	Lampyrinae	JN290381.1
*Aspisoma* sp.	Lampyridae	Lampyrinae	EU009322.1
*Lucidinaaccensa*	Lampyridae	Lampyrinae	AB608771.1
*Lucidinakotbandia*	Lampyridae	Lampyrinae	FJ462784.1
*Lucidotaatra*	Lampyridae	Lampyrinae	HQ984304.1
*Photurispensylvanica*	Lampyridae	Photurinae	MF634963.1
*Photurisquadrifulgens*	Lampyridae	Photurinae	HM433520.1
*Bicellonychalividipennis*	Lampyridae	Photurinae	KJ922151.1
*Bicellonychawickershamorum*	Lampyridae	Photurinae	EU009302.1
*Pristolycus* sp.	Lampyridae	Luciolinae	MK292099.1
*Sclerotiaflavida*	Lampyridae	Luciolinae	KP763460.1
*Sclerotiaaquatilis*	Lampyridae	Luciolinae	KP763466.1
*Pygolucioladunguna*	Lampyridae	Luciolinae	MT106243.1
*Pygoluciolaqingyu*	Lampyridae	Luciolinae	MK292093.1
*Curtosbilineatus*	Lampyridae	Luciolinae	NC_044789.1
*Curtoscostipennis*	Lampyridae	Luciolinae	AB608764.1
*Absconditaterminalis*	Lampyridae	Luciolinae	NC_044776.1
*Absconditaanceyi*	Lampyridae	Luciolinae	NC_039706.1
*Emeiapseudosauteri* 1	Lampyridae	Luciolinae	MN722654.1
*Emeiapseudosauteri* 2	Lampyridae	Luciolinae	OK103803
*Emeiapulchra*	Lampyridae	Luciolinae	OK144132
*Luciolaitalica*	Lampyridae	Luciolinae	KM448530.1
*Asymmetricatacircumdata*	Lampyridae	Luciolinae	NC_032062.1
*Drilasteraxillaris*	Lampyridae	Ototretinae	AB608756.1
*Drilasterokinawensis*	Lampyridae	Ototretinae	AB608758.1
*Stenocladiusyoshikawai*	Lampyridae	Ototretinae	AB608759.1
*Lamprigerayunnana*	Lampyridae	*incertae_sedis*	MG200082.1
*Cyphonocerusmarginatus*	Lampyridae	Cyphonocerinae	AB608754.1
*Rhagophthalmuslufengensis*	Rhagophthalmidae	–	DQ888607.1
*Rhagophthalmusohbai*	Rhagophthalmidae	–	AB608775.1

## ﻿Results

### ﻿Phylogenetic analysis

The *cox1* barcode sequences of *E.pseudosauteri* and *E.pulchra* share a 94% sequence identity over the 658 bp segment. The phylogeny constructed from *cox1* of fireflies showed three main clades corresponding to Lampyrinae, Photurinae and Luciolinae (Fig. 2). *Emeiapseudosauteri* was recovered as sister to *E.pulchra* Zhu & Zhen sp. nov. within the subfamily Luciolinae, with strong support (100%).

### ﻿Taxonomic treatment

#### ﻿ Emeia

Taxon classificationAnimaliaColeopteraLampyridae

Fu, Ballantyne & Lambkin, 2012

281FA718-B336-5F10-BF93-125E93FBD223

##### Type species.

*Emeiapseudosauteri* Geisthardt, 2004 (designated by Fu, Ballantyne and Lambkin 2012).

##### Diagnosis

**(based on adult male).***Emeia* belongs to a group of Luciolinae in which the males have aedeagal parameres widely visible beside the phallus (Ballantyne et al. 2013). *Emeia* differs from *Aquaticawuhana* Fu & Ballantyne, 2010 and *A.lateralis* Motschulsky, 1860, which have black marks on the pronotum (Fu et al. 2010). *Emeia* is distinguished from *Curtos* Motschulsky, 1845, as the species in *Curtos* have a distinctive longitudinal elytral humeral carina and parameres unequal in length (Fu et al. 2012). *Emeia* is closely related to *Pygoluciola* based on our *cox1* phylogeny (Fig. 2), but the two genera can be distinguished by the shape of the pronotum, with median anterior margin gently rounded or slightly medianly emarginate in *Pygoluciola* (Ballantyne and Lambkin 2006) versus lateral margins of pronotum almost parallel in *Emeia*.

##### Description

**Figures 3–4.**
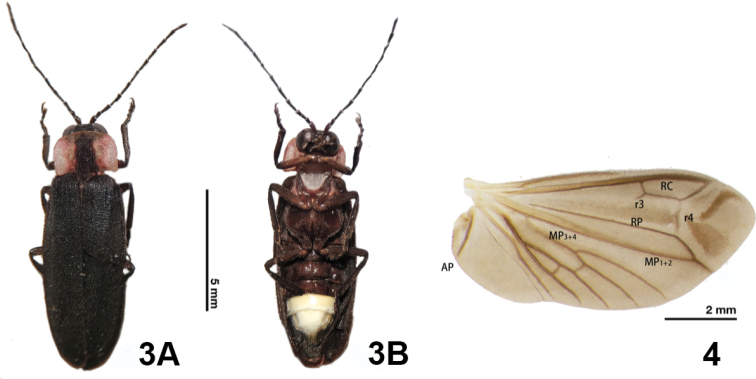
*Emeiapulchra* Zhu & Zhen sp. nov., male **3** habitus of holotype **A** dorsal view **B** ventral view **4** right wing, dorsal view. Scale bars: 5 mm (**3**); 2 mm (**4**).

**(based on adult male).** Body length 6.5–10.5 mm. Body width 2.7–4.0 mm. Integument black or dark brown, with a narrow (e.g., in *E.pulchra*, see Fig. 3A) or thick (e.g., in *E.pseudosauteri*, see Fig. 8A) black stripe on pronotum.

***Head*.** Hypognathous; head depressed between eyes, eyes exposed in front of pronotum; antennae filiform, with 11 antennomeres (Figs 3B, 8B).

***Thorax*.** Pronotum in dorsal view appearing pink-red or orange-red, with a black median stripe, lateral margins almost parallel (Figs 3A, 8A); surface of elytra smooth, longitudinal carina absent (Figs 3A, 8A); legs long and straight, no femora or tibiae swollen or curved (Figs 3B, 8B).

***Abdomen*.** V2–V5 dark brown or black. Light organs present in V6 and V7, entirely occupying V6; V7 semitransparent (Figs 3B, 8B).

***Male genitalia*.** Trilobate, parameres extending ~0.14 mm (n = 3) beyond phallus; both parameres equal in length (Figs 6A, 12A).

#### ﻿ Emeia pulchra

Taxon classificationAnimaliaColeopteraLampyridae

Zhu & Zhen sp. nov.

48F99E78-A1CE-5C9E-8F7E-C5FEE96CB314

https://zoobank.org/45330183-64CB-45CE-A2E4-7E013ECECB00

Figs 3–4 , 5 , 6

##### Diagnosis

**(based on adult male).** The new species can be differentiated from *E.pseudosauteri* Fu, Ballantyne & Lambkinby the elytron, hindwing venation and aedeagus. In fresh specimens, the elytral apices are black in *E.pulchra* (Fig. 3), but with a narrow orange stripe in *E.pseudosauteri* (Fig. 8). In the male hindwing, the upper vein of the MP_3+4_ venation in *E.pulchra* reaches the margin of the hind wings without forks (n=2) (Fig. 4). In *E.pseudosauteri*, the upper vein of MP_3+4_ forks and reaches the margin of the hind wings (n=2) (Fig. 10). The aedeagus in *E.pulchra* is approx. 3 times as long as wide (length 1.77 mm: width 0.58 mm) (Fig. 6A), versus approx. 2 times as long as wide (length 1.66 mm: width 0.84 mm) in *E.pseudosauteri* (Fig. 12A).

##### Description.

**Male**: BL 10.0–10.4 mm; BW 3.5–3.7 mm (three individuals).

***Head*.** Antennae filiform, black, almost 2/3 as long as body length; antennomere 1 cone-shaped; 2 short and cylindrical; 3 to 10 compressed, not bifurcate; 11^th^ antennomere almost 1.5 times longer than 10^th^, slightly dilated from base to apex. Concave between eyes dorsally in cross section, both eyes occupying about 2/3 width of whole head in ventral view. Eyes spherical, so that head cannot fully contract into pronotum. Mouthparts fully developed, clypeolabral suture flexible, outer edges of labrum reaching inner edges of closed mandibles.

***Thorax*.** Scutellum black and slightly emarginate distally. Elytra elongated, dark brown to black, apices not deflexed in dorsal view, sides slightly convex. Hind wing well developed, r3 half the length of r4 (Fig. 4). Legs long and straight, without swelling on any part, dark brown to black, with dense white hairs.

**Figure 5.**
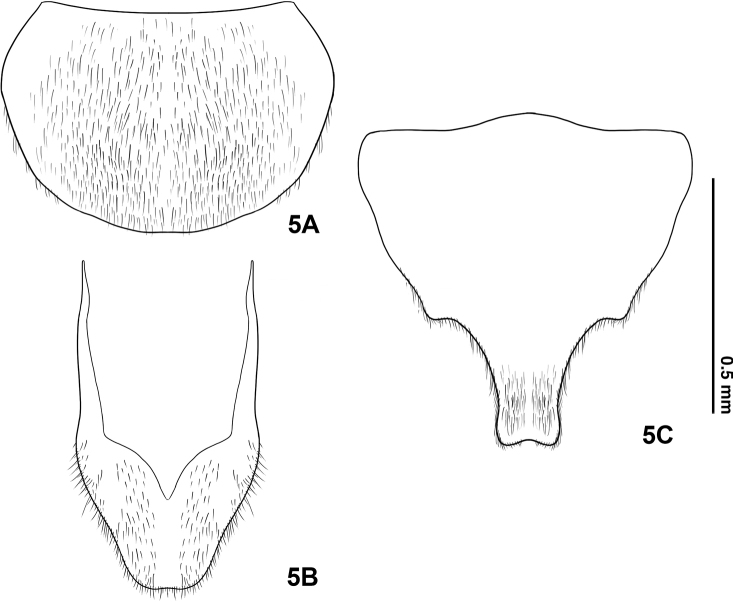
Male abdominal ventrites (V) and tergites (T) of *Emeiapulchra* Zhu & Zhen, sp. nov. **A**T7**B**T8**C**V7. Scale bar: 0.5 mm.

***Abdomen*.** Dark brown, ventrites gradually diminishing in length posterad. Light organs yellow-white, occupying almost all of V6 and half of V7, not reaching to posterior edges of V7. V6 and V7 rounded laterally (Fig. 5), posterior half of V7 not arched in dorsal view, abruptly narrowed to truncate posterior apex, apex emarginate (Fig. 5C). T7 rounded, without anterolateral corners (Fig. 5A); T8 symmetrical with concealed anterolateral arms, widest across middle with lateral margins subparallel-sided in anterior half, tapering evenly in posterior half to a rounded and partly truncate posterior margin (Fig. 5B). Abdominal spiracles on lateral edges of each abdominal segments. EL/EW = 4.7–4.8; EL/PL= 4.7–5.0 (n=3).

**Figure 6.**
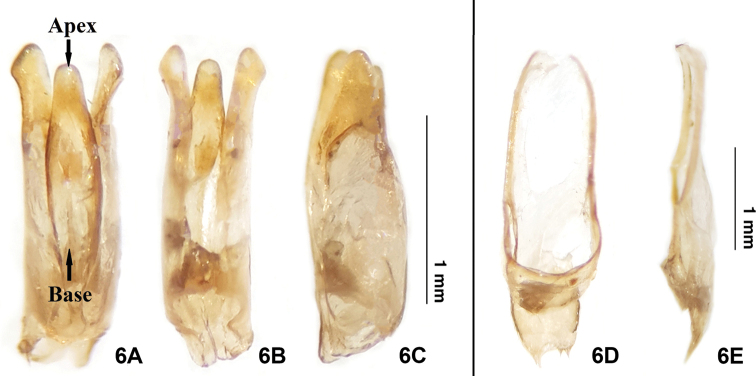
Aedeagusof *Emeiapulchra* Zhu & Zhen sp. nov. **A** dorsal view **B** ventral view **C** lateral view. Aedeagal sheath of *E.pulchra***D** dorsal view **E** ventral view. Scale bar: 1 mm.

***Male genitalia*** (Fig. 6): Aedeagal sheath (T9, T10, S9) (Fig. 6D, E) 3.15 mm long; anterior half of sternite broad, apically rounded; tergite without protrusion along posterior margin of T9. Aedeagus (Fig. 6A–C) 1.61 mm long. Phallus short (~1.2 mm) and thick, broadest at midlength, becoming thinner at apex and base, parameres (lateral lobes) extending about 0.14 mm beyond phallus. Parameres robust, subparallel-sided, symmetrical, with blunt preapical lateral expansion.

**Figure 7.**
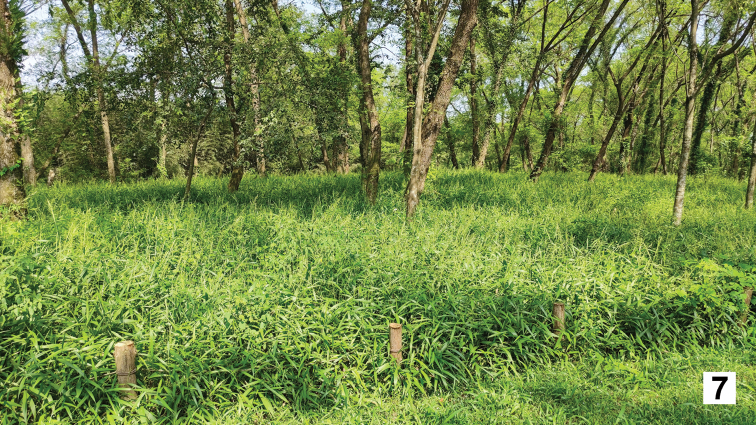
Habitat of *Emeiapulchra* Zhu & Zhen sp. nov. in Jiulong National Wetland Park.

##### Etymology.

The specific name *pulchra* refers to the bright pronotum coloration.

##### Holotype.

China • 1♂; Zhejiang, Lishui; 28°37.56'N, 119°49.7'E; H: 60 m, 2. IV. 2020; Chengqi Zhu leg.; ‘HOLOTYPE (red), ♂, *Emeiapulchra* sp. nov., det. Zhu, Zhen, 2021’ (Westlake University).

##### Paratype.

China • 1♂; Zhejiang, Lishui; 28°37.56'N, 119°49.7'E; H: 60 m, 2. IV. 2020; Chengqi Zhu leg.; ‘PARATYPE (yellow), ♂, *Emeiapulchra* sp. nov., det. Zhu, Zhen, 2021’ (Westlake University).

##### Distribution.

China: Zhejiang Province.

##### Habitat and occurrence.

The males were found in an open forest of mainly Chinese wingnut, of the family Juglandaceae [*Pterocaryastenoptera* C. DC.] (Fig. 7). The floor of the *Emeiapulchra* habitat was covered with a lush herbaceous layer 20–30 cm high.

There are many terrestrial snails and slugs in this habitat, which may be potential food for *Emeiapulchra* larvae. Combining descriptions from local people and our field observations, adult fireflies are usually observed mid-March. The protection of fireflies has been supported by the Lishui government and Jiulong National Wetland Park management departments, and this area has been protected as Jiulong National Wetland Park (Fig. 7). Fan (2019) reported that the population size of *E.pulchra* has increased from 2014 to 2019 with the protection efforts.

##### Behavioral remarks.

There are two obvious luminous bands at the terminal end of the adult male abdomen. The two bands both emit intermittent bright light during courtship. The male courtship behavior usually starts at 19:00 (approximately 1h after sunset), and peaks at about 20:30. Adult males rest on higher herbs and emit yellow and green flashing light. Males are reluctant flyers; the distance of each flight ranges from 0.5 to 5 m.

**Figures 8–11.**
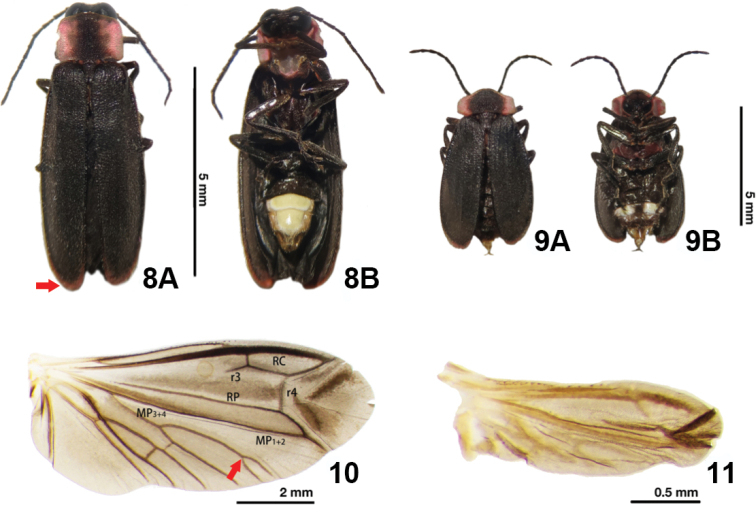
*Emeiapseudosauteri* Fu, Ballantyne & Lambkin, 2012. Male and female **8** habitus of male **A** dorsal view. Arrow highlights narrow orange stripe on elytral apices. The color appears darker in this photo, but it is orange and easily seen in both dried and fresh samples **B** ventral view **9** habitus of female. **A** dorsal view **B** ventral view **10** right wing of male. Dorsal view. Arrow points to wing venation, which differs between the two *Emeia* species **11** right wing of female. Dorsal view. Scale bars: 5 mm (**8, 9**); 2 mm (**10**); 0.5 mm (**11**).

#### ﻿ Emeia pseudosauteri

Taxon classificationAnimaliaColeopteraLampyridae

(Geisthardt 2004)

9DA45D79-547B-5ADB-9222-5C5E87FBF0C3

Figs 8–11 , 12

Emeia pseudosauteri (Geisthardt 2004). *Zootaxa* (3403), 1–53. TL: ‘Mt. Tian Tai, Sichuan Province, China’.

##### Specimens examined.

**Figure 12.**
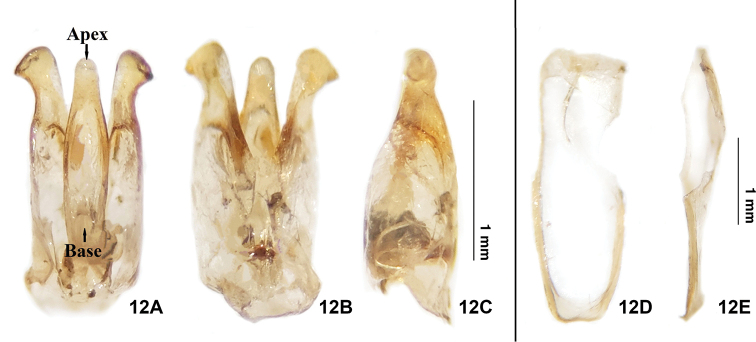
Aedeagus of *Emeiapseudosauteri.***A** dorsal view **B** ventral view **C** lateral view. Male aedeagal sheath of *E.pseudosauteri***D** dorsal view **E** ventral view. Scale bars: 1 mm.

China: 6♂♂,1♀, Sichuan, Mt. Tian Tai, 3.IV. 2021, Chengquan Cao leg. We herein examined specimens of *E.pseudosauteri* from Mt. Tian Tai (the type locality), and their identity was further verified using *cox1* barcode sequences (Fig. 2) and morphological examination (Figs 8–12).

**Figure 13.**
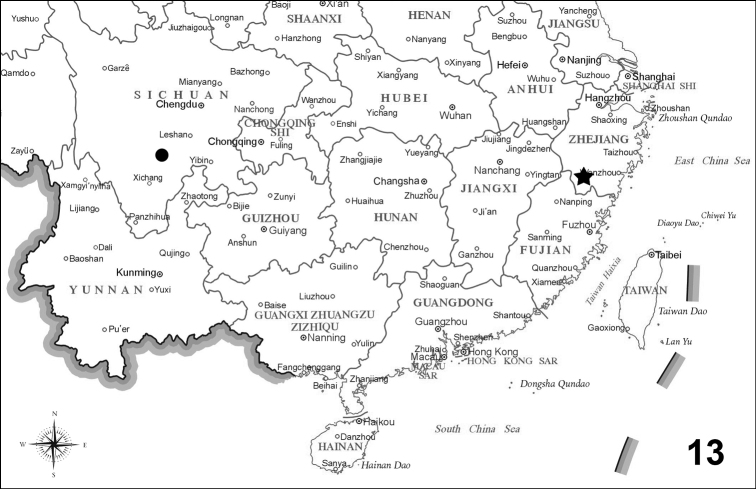
Distribution map of the genus *Emeia* in China. The black star indicates *E.pulchra* Zhu & Chen sp. nov., the black dot *E.pseudosauteri* (map of China from: http://bzdt.ch.mnr.gov.cn/).

### ﻿Key to species (adult males)

**Table:** 

1	The elytral apices have a narrow orange stripe in both fresh and dried specimens; upper vein of MP_3+4_ forked and reaching edge of hind wing (Fig. 10); phallus and parameres broad, 2 times as long as wide (Fig. 12A)	***E.pseudosauteri* Fu, Ballantyne & Lambkin**
–	The elytral apices are black in fresh and preserved specimens (Fig. 3A); upper vein of MP_3+4_ reaching margin of hind wings, but without forks (Fig. 4); phallus and parameres slender, 3 times as long as wide (Fig. 6A)	***E.pulchra* Zhu & Zhen, sp. nov.**

## ﻿Discussion

In this study, we summarized the diagnostic features of the genus *Emeia*. *Emeiapulchra* Zhu & Zhen, sp. nov. is morphologically similar to *E.pseudosauteri* Fu, Ballantyne & Lambkin, 2012 from Sichuan Province. However, we found differences in the antennal length and body size between the two species. The body size of a species may vary due to nutrition and environmental factors, so we did not include size in the diagnosis to the new species. The antenna of male *E.pulchra* (Fig. 3) is narrower than that of *E.pseudosauteri* (Fig. 8) in lateral view. Females of *E.pseudosauteri* have body length about 2/3 of that of the male and have normal elytra (Fig. 9), but their hind wings are small and shrunken, about 1/4 length of the male hind wings (Figs 10, 11). In the male, we found that the hind wing of *E.pseudosauteri* was relatively narrower and longer than that of *E.pulchra*. The elytral apice has a narrow orange stripe in both fresh and dried specimens of *E.pseudosauteri*, whereas it is black in *E.pulchra* (in three *E.pulchra* and six *E.pseudosauteri* examined). The observed body size of *E.pseudosauteri* (BL 6.6–7.2 mm; BW 2.7–2.9 mm; six individuals measured) was smaller than for *E.pulchra* (BL 10.0–10.4 mm; BW 3.5–3.7 mm; three individuals measured). In the male genitalia, the aedeagus of *E.pulchra* (Fig. 6A) is narrower than that of *E.pseudosauteri* (Fig. 12A), and the parameres are less curved (Figs 6B, 12B). In addition, the new species is only known from S. Zhejiang, whereas *E.pseudosauteri* is only found 1600 km westward, in the Sichuan Province (Fig. 13).

The “barcode region” of *cox1* is often used as an aid to new species’ identification and distinction from close relatives in the Barcode of Life Data system (Ratnasingham and Hebert 2007; Lin et al. 2009). Currently, this method has been widely and successfully used to identify closely-related species and conspecific individuals. Our *cox1* gene tree recovered the major subdivisions within Lampyridae, including Lampyrinae, Photurinae and Luciolinae. This tree is consistent with recent studies using 436 loci (Martin et al. 2019) or 15 mitochondrial genes (Chen et al. 2019), and supports that the placement of *Emeia* in Luciolinae (Fig. 2). Both the *cox1* tree and morphology support *E.pulchra* as the closest sister species of *E.pseudosauteri*.

## Supplementary Material

XML Treatment for Emeia

XML Treatment for Emeia pulchra

XML Treatment for Emeia pseudosauteri

## References

[B1] Ballantyne LA Lambkin CL (2006) A phylogenetic reassessment of the rare SE Asian firefly genus *Pygoluciola* Wittmer (Coleoptera: Lampyridae: Luciolinae). The Raffles Bulletin of Zoology 54: 21–48.

[B2] Ballantyne L Fu XH Lambkin C Jeng ML Faust L Wijekoon W Li DQ Zhu TF (2013) Studies on South-east Asian fireflies: *Abscondita*, a new genus with details of life history, flashing patterns and behaviour of *Abs.chinensis* (L.) and *Abs.terminalis* (Olivier) (Coleoptera: Lampyridae: Luciolinae). Zootaxa 3721(1): 1–48. 10.11646/zootaxa.3721.1.1 26120657

[B3] Chen X Dong Z Liu G He J Zhao R Wang W Peng Y Li X (2019) Phylogenetic analysis provides insights into the evolution of Asian fireflies and adult bioluminescence. Molecular Phylogenetics and Evolution 140: e106600. 10.1016/j.ympev.2019.106600 31445200

[B4] Douglas HB (2017) World reclassification of the Cardiophorinae (Coleoptera,Elateridae), based on phyloogenetic analyses of morphological characters. ZooKeys 655: 1–130. 10.3897/zookeys.655.11894 PMC5345379 28331397

[B5] Fan L (2019) Inverstigation and Analysis of Insect Resources in Lishui Jiulong National Wetland Park. In: Zhejiang A&F University, Zhejinag, China, 1–35.

[B6] Folmer O Black M Hoeh W Lutz R Vrijenhoek R (1994) DNA primers for amplification of mitochondrial cytochrome c oxidase subunit I from diverse metazoan invertebrates. Molecular Marine Biology and Biotechnology 3: 294–299. 7881515

[B7] Fu XH Ballantyne L Lambkin CL (2010) *Aquatica* gen. nov. from mainland China with a description of *Aquaticawuhana* sp. nov. (Coleoptera: Lampyridae: Luciolinae). Zootaxa 2530(1): 1–18. 10.11646/zootaxa.2530.1.1

[B8] Fu XH Ballantyne L Lambkin CL (2012) *Emeia* gen. nov., a new genus of Luciolinae fireflies from China (Coleoptera: Lampyridae) with an unusual trilobite-like larva, and a redescription of the genus *Curtos* Motschulsky. Zootaxa 3403(1): 1–53. 10.11646/zootaxa.3403.1.1

[B9] Geisthardt M (2004) New and known fireflies from Mount Emei (China) (Coleoptera: Lampyridae). Mitteilungen des Internationalen entomologischen Vereins 29: 1–10.

[B10] Lin S Zhang H Hou Y Zhuang Y Miranda L (2009) High-level diversity of dinoflagellates in the natural environment, revealed by assessment of mitochondrial *cox1* and *cob* genes for dinoflagellate DNA barcoding. Applied and Environmental Microbiology 75(5): 1279–1290. 10.1128/AEM.01578-08 19114529 PMC2648164

[B11] Martin GJ Stanger-Hall KF Branham MA Da Silveira LF Lower SE Hall DW Li XY Lemmon AR Lemmon EM Bybee SM (2019) Higher-level phylogeny and reclassification of Lampyridae (Coleoptera: Elateroidea). Insect Systematics and Diversity 3(6): 11. 10.1093/isd/ixz024

[B12] Ratnasingham S Hebert PD (2007) BOLD: The Barcode of Life Data System (http://www.barcodinglife.org). Molecular Ecology Notes 7(3): 355–364. 10.1111/j.1471-8286.2007.01678.x PMC1890991 18784790

[B13] Tamura K Stecher G Peterson D Filipski A Kumar S (2013) MEGA6: Molecular Evolutionary Genetics Analysis Version 6.0. Molecular Biology and Evolution 30(12): 2725–2729. 10.1093/molbev/mst197 24132122 PMC3840312

